# Exosomal miR-328 originated from pulmonary adenocarcinoma cells enhances osteoclastogenesis via downregulating Nrp-2 expression

**DOI:** 10.1038/s41420-022-01194-z

**Published:** 2022-10-03

**Authors:** Chengcheng Zhang, Jingru Qin, Lu Yang, Zhiyao Zhu, Jia Yang, Wan Su, Haibin Deng, Zhongqi Wang

**Affiliations:** 1 Department of Medical Oncology, Longhua Hospital affiliated with Shanghai University of Traditional Chinese Medicine, 725 Wanpingnan Road, Shanghai, 200032 China; 2 Cancer Institute, Longhua Hospital affiliated with Shanghai University of Traditional Chinese Medicine, 725 Wanpingnan Road, Shanghai, 200032 China

**Keywords:** Small-cell lung cancer, Bone metastases

## Abstract

Osseous metastases of pulmonary carcinoma and the detailed mechanisms remain unclear, and the effects of exosomes (Exos) originated from pulmonary adenocarcinoma cells in this process have received a lot of attentions. Our study revealed that the Exos secreted from A549 cells (A549-Exos) enhanced osteoclastogenesis and osseous resorption in vitro. In addition, A549-Exos showed a targeted effect on bones to enhance osseous resorption in vivo. A549-exosomal miR-328 enhanced osseous resorption via downregulating neuropilin 2 (Nrp-2) expression, and A549-Exos miR-328 inhibitors suppressed osseous resorption in vivo. Therefore, A549-exosomal miR-328 enhances osteoclastogenesis via downregulating Nrp-2 expression, thus A549-Exos ^miR-328 inhibitors^ can be used as a potential nanodrug for treating osseous metastases.

## Introduction

Bones are the mostly frequent sites of metastasis in patients with pulmonary carcinoma [1], and most osseous metastases of pulmonary carcinoma can be classified as osteolytic osseous metastases [2], which will lead to pain, pathological fracture, instable spine, compressive myelopathy and hypercalcaemia, thus resulting in a decline in the quality of life [3, 4]. However, the proteolytic enzymes in pulmonary carcinoma cells can’t cause osteolytic metastases in a direct manner. Instead, the pulmonary carcinoma cell-derived circulating miRNAs and exosomes (Exos) play important roles by activating osteoclasts [5, 6]. Thus, we focused on the effects of Exos excreted from pulmonary carcinoma in osteoclast differentiation.

Exos are nanoscale vesicles with a diameter of 80–150 nm. Exos contain mRNAs, miRNAs [7], lipids [8] and proteins [9]. It been reported that Exos exert an effect on cellular communication via transferring proteins [10], bioactive lipids and miRNAs [11]. The Exos originated from pulmonary adenocarcinoma cells have received a lot of attentions due to their effects on osseous metastases and prognosis [12]. Furthermore, Exos may potentially be applied as biomarkers, and carriers of nanoparticle drugs and antitumor drugs. In recent years, multiple studies revealed that exosomal miRNAs exerted important effects on the abilities of proliferating, invading and metastasizing of tumour cells [13]. Therefore, finding the exosomal miRNAs are critical for treating osseous metastases, and exosomal miRNAs can be utilized as a novel target for treating osseous metastases [14].

Our study revealed that the Exos secreted from A549 cells (A549-Exos) are important extracellular vesicles in osteoclast differentiation and osseous metastases. Furthermore, we found that A549-Exos enhanced osseous resorption in vitro and in vivo, and a further investigation on its mechanism revealed that A549-exosomal miR-328 enhanced osseous resorption via downregulating neuropilin 2 (Nrp-2) expression. Therefore, A549-Exos were treated with miR-328 inhibitors, and then it was found that osseous resorption was prevented by injecting A549-Exos^miR-328 inhibitors^. Therefore, we focused on the effects of A549-Exos on osseous resorption in present study.

## Results

### Characteristics of the isolated A549-Exos

**Fig. 1:**
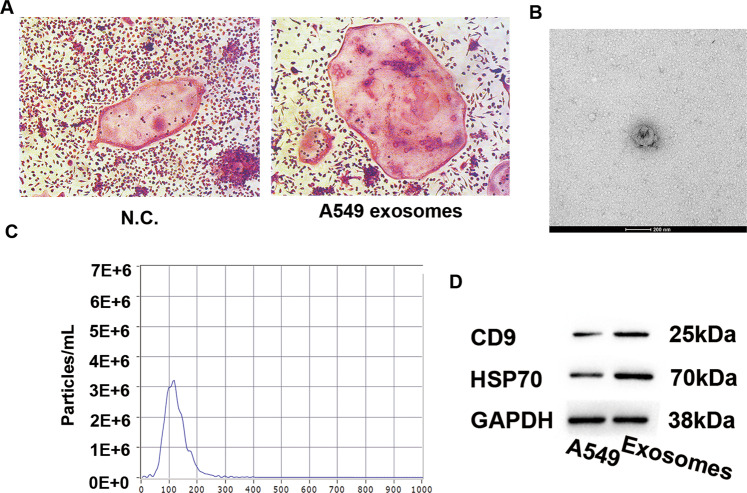
Characteristics of the isolated A549-Exos. **A** Representative images of TRAP-stained osteoclasts. Scale bar: 100 μm. **B** Representative TEM image of A549-Exos (scale bar = 200 nm). **C** Results of nanoparticle tracking analysis reflecting the size distribution of A549-Exos. **D** Detection of A549-Exos surface markers (CD9 and HSP70) by a Western blot analysis.

For exploring the mechanism of action of pulmonary adenocarcinoma cells in osteoclastogenesis, it was first determined whether pulmonary adenocarcinoma cells could affect the differentiation of osteoclasts. RAW 264.7 cells were co-cultivated with A549 cells in a Transwell system according to previous studies [15, 16]. The differentiation of osteoclasts was enhanced when co-cultivated with A549 cells (Figs. 1A, S1A). Then, the transmission electron microscopy, dynamic light-scattering assay and Western-blot assay were conducted to characterize the A549-Exos. A549-Exos showed a cup-like or spherical shape, with a primary diameter range of 80–150 nm (Fig. 1B, C). The Western blot assay revealed that the Exos marker proteins such as CD9 and TSG101 were expressed in A549-Exos (Figs. 1D, S1B). Thus, these data suggest that osteoclastic differentiation was enhanced when cocultured with A549 cells and that A549-Exos showed characteristic features of Exos.

### A549-Exos enhanced osteoclast differentiation and osseous resorption in vitro

**Fig. 2:**
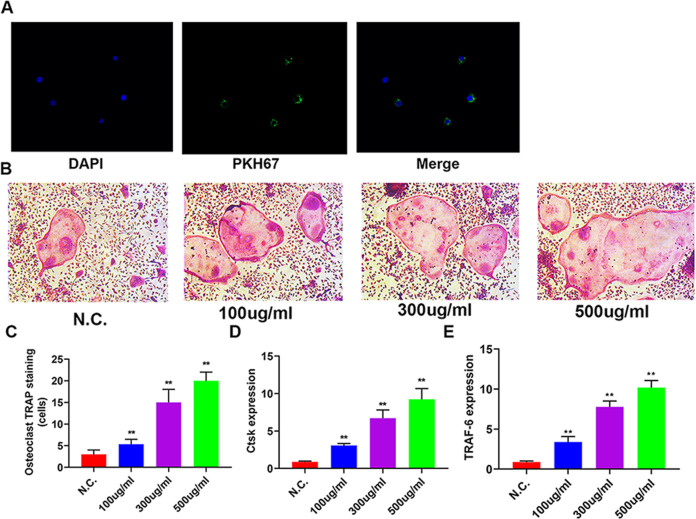
A549-Exos suppressed osteoclast differentiation and osseous resorption in vitro. **A** Fluorescence microscopy analysis of RAW 264.7 cells treated with DiO-labelled A549-Exos for 2 h. **B** TRAP staining images of mature osteoclasts processed with different concentrations of A549-Exos. Scale bar: 100 μm. **C** Quantitative assessment of the positive TRAP-stained osteoclasts in Fig. 2B (**P < 0.01). **D**–**E** Relative expression levels of Ctsk and TRAF-6 mRNA in Fig. 2B (**P < 0.01).

To investigate the effect of A549-Exos on osteoclastogenesis in vitro, A549-Exos were labelled with the lipophilic dye Dio, and whether A549-Exos could be transported to RAW 264.7 cells was assessed. Fluorescence microscopy revealed that A549-Exos could be successfully absorbed by RAW 264.7 cells (Fig. 2A). Then, RAW 264.7 cells were treated by A549-Exos or PBS in same volume. Osteoclastic differentiation was up-regulated along with the increase in the concentration of A549-Exos (Fig. 2B, C). Furthermore, we found that the mRNA expression of Ctsk and Traf-6 was increased by A549-Exos in comparison with the negative control on the 7th day (Fig. 2D, E). Taken together, the above results indicate that A549-Exos enhance osteoclast differentiation in vitro.

### A549-Exos target to bone in vivo

**Fig. 3:**
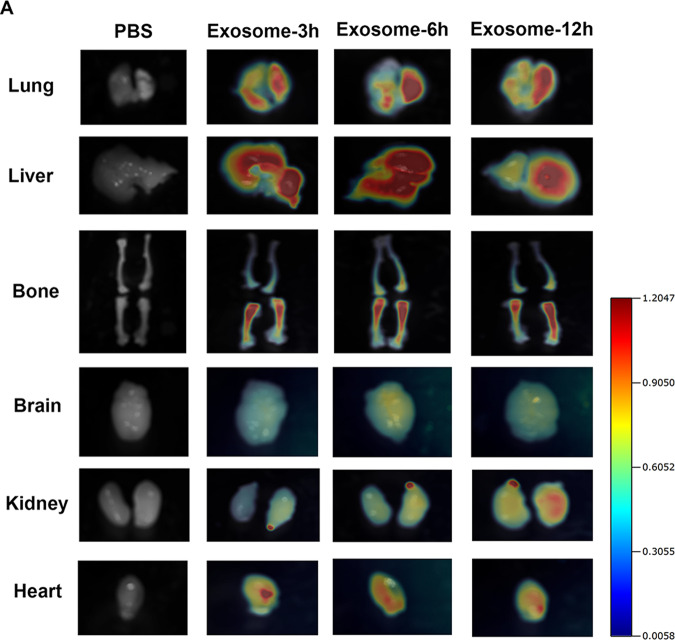
A549-Exos target bone in vivo without toxicity. **A** Fluorescence molecular tomography, images of near-infrared fluorescence signals in organs harvested from mice treated with PBS or A549-Exos for different durations (3 h, 6 h, and 12 h).

For exploring the biodistribution of A549-Exos in vivo, the tissue distribution of DID-labelled A549-Exos (A549-Exos-DID) was examined by a fluorescence molecular tomography (FMT) imaging system. 8-week-old male mice were intravenously administrated with A549-Exos-DID via the tail vein for 3, 6 or 12 h. At three hours after the treatment, the major fluorescent signals were observed in the liver and lungs, and the signals could be measured in the limbs. Six hours later, the major fluorescent signals still accumulated within the liver and lung. Significantly, the fluorescent signals in the limbs were enhanced but still weaker than those in the liver and lung. Twelve hours later, fluorescent signals could still be detected in the limbs, and the fluorescent signals faded in the liver and lungs. Fluorescent signals were rarely detected in the kidney (Fig. 3A). Furthermore, the HE staining indicated no obvious significant histologic differences in the heart, liver, lung, and kidney between the different groups, indicating that A549-Exos are well tolerated, with limited to no acute and systemic toxicity (Fig. S2). Taken together, A549-Exos could target to osseous tissues in vivo without dose-limiting toxicity.

### A549-Exos suppressed osseous formation in vivo

**Fig. 4:**
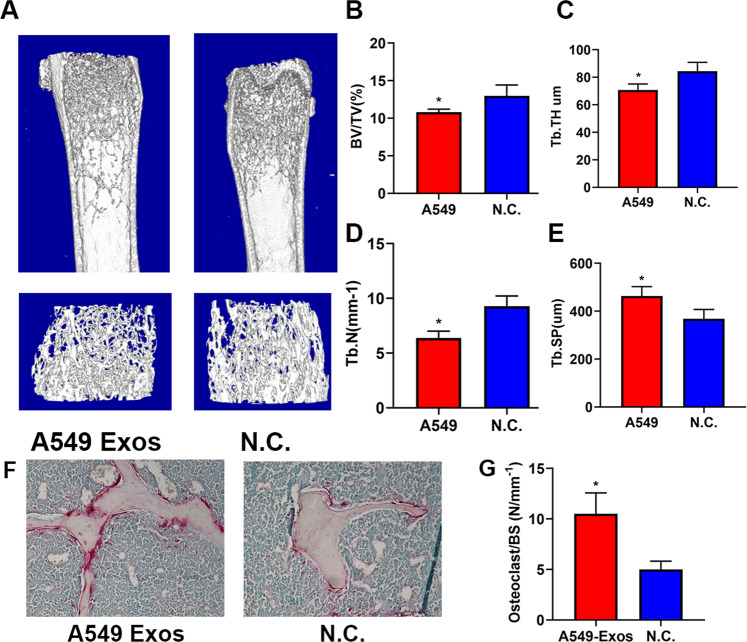
A549-Exos inhibited osseous formation in vivo. **A** microCT images of femora harvested respectively from A549-Exos-treated mice and the negative control group (*n* = 8 in each group). Scale bar: 100 μm. **B**–**E** Quantitative comparison of the trabecular microarchitecture between the A549-Exos group and the negative control group. **F** TRAP staining images of femora harvested respectively from A549-Exos-treated mice and the negative control group. **G** Quantitative comparison of osteoclasts per osseous surface between different groups. BV/TV trabecular bone volume per tissue volume, Tb. Th trabecular thickness, Tb. Sp trabecular separation, Tb. N trabecular number. (*n* = 8 in each group) (*P < 0.05).

For investigating the effect of A549-Exos in vivo, 8-week-old male mice were intravenously administrated with A549-Exos or PBS in same volume via the tail vein three times per week for 12 weeks. Micro-CT indicated an obviously decrease in the volume, number and thickness of trabecular bones and an increased trabecular separation in the mice receiving treatment with A549-Exos in comparison with those in the controls (Fig. 4A, B–E). Furthermore, the TRAP assay revealed that the counts of osteoclasts on the trabecular bone surface were greater in the A549-Exos groups than in the negative controls (NC), indicating that A549-Exos act as an agonist in osteoclast differentiation, this result was in consistency with that of the in vitro study (Fig. 4F, G). The present study indicate that A549-Exos can enhance osseous resorption in vivo.

### A549-Exos miR-328 inhibited osseous formation via downregulating Nrp-2 expression

**Fig. 5:**
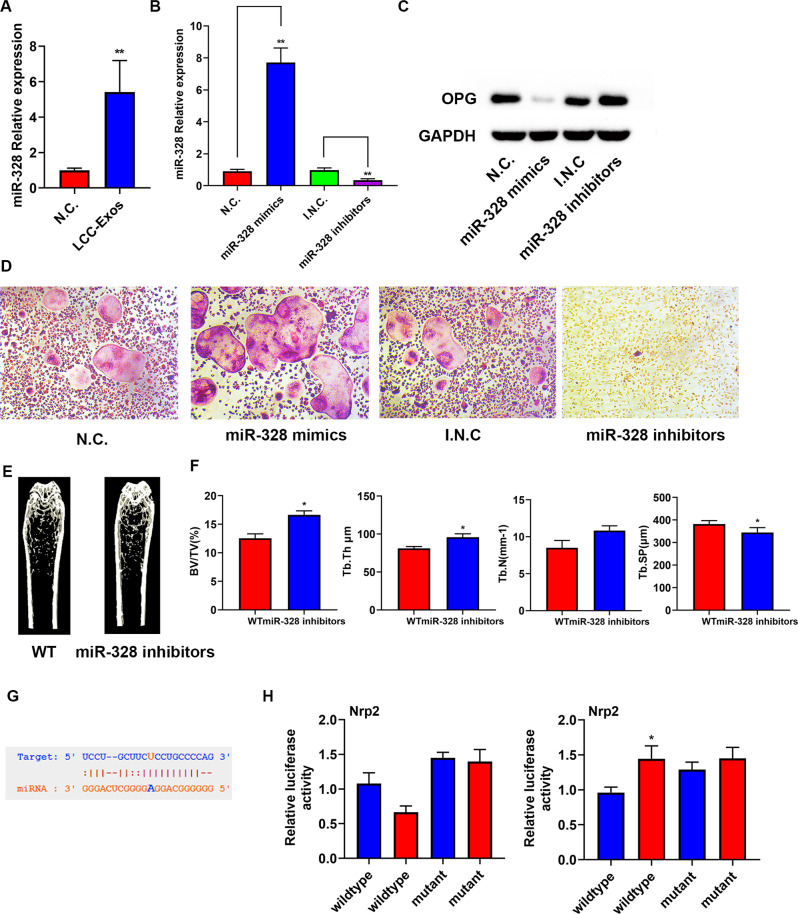
A549-Exoso miR-328 enhanced osseous resorption via downregulating Nrp-2 expression. **A** Relative miR-328 expression in N.C. and LCC-Exos. **B** miR-328 levels in N.C., miR-328 mimics, I.N.C. and miR-328 inhibitors were measured by qRT–PCR. **C** Western blot (WB) assay of OPG and GAPDH in RAW 264.7 cells receiving transfection with miR-328 mimics and miR-328 or their respective negative controls. **D** TRAP staining images of mature osteoclasts treated with N.C., miR-328 mimics, I.N.C. and miR-328 inhibitors. **E** Representative micro-CT images of femora from different groups. **F** Quantitative comparison of the trabecular microarchitecture between different groups. **G** Nrp-2 might be a target of miR-328. **H** RAW 264.7 cells received transfection with a luciferase reporter, which carried WT or MUT-Nrp2 3′-UTR in the miR-328 mimics (**H** left) and miR-328 inhibitors (**H** right). *P < 0.05, **P < 0.01. The data are an average of at least three independent experiments.

Exos may concentrate large numbers of miRNAs, which can regulate gene expression by binding 3′-untranslated regions. MiR-328 has been reported to promote osteoclast differentiation [17]. Thereafter, the expression of miR‐328 in the Exo was detected, it turned out that the expression of miR-328 was obviously stronger in A549-Exos than in TC-1-cell-derived Exos according to previous studies [18, 19] (Fig. 5A).

RAW 264.7 cells underwent transfection with miR-328 mimics or inhibitors for exploration on osteoclastogenesis regulated by miR-328 in vitro. The expressional level miR-328 was obviously increased by miR-328 mimics and reduced by miR-328 inhibitors (Fig. 5B). In addition, the overexpressed miR-328 could suppress the endogenous OPG protein expression (Fig. 5C). In consistency with the expressiona level of OPG proteins, it was revealed that TRAP staining was deepened by miR-328 mimics and weakened by miR-328 inhibitors (Figs. 5D, S3). Overall, these data show that miR-328 can enhance osteoclast differentiation in vitro.

To determine whether osseous resorption is regulated by miR-328 in vivo, 8-week-old male mice were intraveneously adminstrated with miR-328 inhibitors or their negative controls via the tail vein, three times per week for 8 weeks. The volume, number, and thickness of the trabecular bones were increased and the trabecular separation was reduced in the mice receiving treatment with the miR-328 inhibitors compared with those in the negative group. These data show that miR-328 can regulate osseous resorption in vivo.

For investigating the target mRNAs of miR-328 in the osteoclast differentiation process, the miRNA-binding site in TargetScan was detected, and the results showed that miR-328 bound to the 3′-UTR of Nrp-2 (Fig. 5G). It was previously reported, that Nrp-2 deficiency can enhance the differentiation of osteoclasts and reduce the number of osteoblasts [20]. Thus, we generated the luciferase reporter constructs, which contained WT or mutated predicted miRNA-binding sites of Nrp-2. Then, RAW 264.7 cells were transfected with WT-Nrp2–3′-UTR or MUT-Nrp2–3′-UTR and miR-328 mimics or inhibitors respectively, and the luciferase activity of the Nrp-2 reporter gene was suppressed using miR-328 mimics. The Nrp-2 reporter gene was promoted using miR-328 inhibitors, but MUT-Nrp2–3′-UTR could prevent this inhibitory effect (Fig. 5H). In addition, RAW 264.7 cells were transfected with Nrp-2 inhibitors or the negative control, and we found that the Nrp-2 inhibitors enhanced TRAP staining compared to the negative control (Fig. S4). To sum up, the above data reveal that miR-328 directly targets to Nrp-2 and inhibits Nrp-2 expression in osteoclast cells.

### A549-Exos^miR-328 inhibitors^ inhibited osseous resorption in vivo

**Fig. 6:**
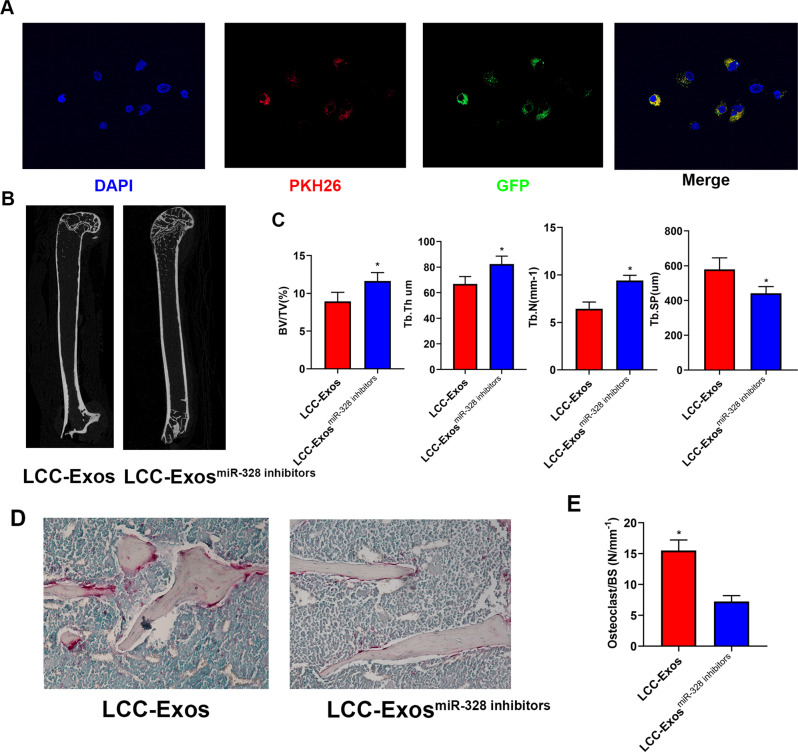
A549-exosmiR-328 inhibitors suppressed osseous resorption in vivo. **A** Fluorescence microscopy analysis of A549 cells treated with DiO-labelled A549-Exos ^miR-328 inhibitors^ for 2 h. **B** Representative micro-CT images of femora harvested respectively from the A549-Exos group and A549-Exos^miR-328 Inhibitors^ group (*n* = 6 in each group). **C** Quantitative comparison of the trabecular microarchitecture between the A549-Exos group and A549-Exos^miR-328 inhibitors^ group (*n* = 8 in each group). **D** TRAP staining images of femora harvested respectively from different groups (*n* = 8 in each group). **E** Quantitative comparison of osteoclasts per osseous surface between different groups. (*P < 0.05).

For exploring the effect of miR-328 in the treatment of osseous metastases, A549-Exos^miR-328 inhibitors^ were generated. A549 cells transfected with miR-328 inhibitor-derived Exos were stained with the lipophilic dyes and assessed to confirm if A549-Exos could carry miR-328 inhibitors to be transported into RAW 264.7 cells. Fluorescence microscopy revealed that A549-Exos^miR-328 inhibitors^ could be successfully absorbed by RAW 264.7 cells (Fig. 6A). Then, 8-week-old male mice were intravenously administrated with A549-Exos or A549-Exos^miR-328 inhibitors^ via their tail veins, three times per week for 12 weeks. The volume, number, and thickness of the trabecular bones were increased and the trabecular separation was reduced in the mice treated with A549-Exos^miR-328 inhibitors^ compared with those in the the A549-Exos group, which showed that A549-Exos^miR-328 inhibitors^ could alleviate bone loss caused by osseous metastases (Fig. 6B, C). In addition, the TRAP assay indicated that the number of osteoclasts on the trabecular bone surface was lower in the A549-Exos^miR-328 inhibitors^ group than that in the A549-Exo group (Fig. 6D, E), revealing an inhibitory role of A549-Exos^miR-328 inhibitors^ in the differentiation of osteoclasts. In conclusion, the above data indicates that an intravenous injection of A549-Exos^miR-328 inhibitors^ suppresses osseous resorption in vivo and alleviates the osteopenia caused by osseous metastases.

## Discussion

The present study revealed that osteoclast differentiation was enhanced when co-cultivated with A549 cells. A549-Exos enhanced osteoclast differentiation in vitro. Furthermore, A549-Exos could target to osseous tissues to enhance osseous resorption in vivo. Moreover, it was found that A549-Exos^miR-328^ enhanced osseous resorption via downregulating Nrp-2 expression. In addition, the A549-Exos^miR-328 inhibitors^ treatment could ameliorate bone loss and prevent pathological fractures in vivo.

Previously, it has been reported that several Exos originated from cancerous cells are home to the osseous microenvironment and can lead to metastasis lesions [21]. As the murine prostate cancer cell line TRAMP-C1 suppresses the ability of RAW 264.7 cells to differentiate into osteoclasts [22], Exos are secreted from non-small-cell lung cancer cells, which can enhance osteoclast differentiation in RAW 264.7 cells through activating EGFR phosphorylation [23], Exos secreted from multiple myeloma cells can promote the surviving and migrating abilities of osteoclast precursors via elevating the expression of CXCR4 [24], and osteoclast precursors and osteoblasts are internalized by extracellular vesicles extracted from PC3 culture medium [25]. In present study, A549 cell-derived exosomal miR-328 was a regulator of osseous resorption, and A549-Exos^miR-328 inhibitors^ could ameliorate bone loss. Thus, A549-Exos could load and deliver small molecule drugs to target to osseous tissues. Since Exos extracted from a set of tumour cells can exert a targeted effect on osseous tissues, we hypothesize that these Exos can also be applied as targeted drug carriers for the treatment of respective primary tumours. This paper may provide a valuable reference for future studies on the relationship between osseous metastases and tumour cell-derived Exos, thus leading to new potential therapeutic targets.

It has been revealed that multiple Exos miRNAs are associated with osseous formation or osseous resorption [26]. For example, Exos miR-141-3p can regulate osteoblast activity to enhance osteoblastic metastases of prostatic cancer [27], overexpressed hsamiR‑940 in tumour Exos ultimately lead to extensive osteoblastic lesions in the resulting tumour [28], and miR-192 cargo within exosomes, such as vesicle transfer, can affect the osseous metastatic colonization [29]. The above miRNAs can also serve as novel targets for the treatment of osseous metastases from pulmonary cancers, which requires further investigation.

## Conclusions

A549-Exos can enhance osteogenesis and osseous resorption in vitro. In addition, A549-Exos shows a targeted effect on bones in vivo and promotes osseous resorption in vivo. A549-exosomal miR-328 enhances osteoclastogenesis via dwonregulating Nrp-2 expression, thus A549-Exos^miR-328 inhibitors^ can be utilized as a novel nanodrug for treating osseous metastases.

## Methods

### Cellular cultivation

A549 cells (American Type Culture Collection, America) were cultivated by DMEM with 10% FBS (Gibco, America). RAW 264.7 cells were cultivated by DMEM medium with 10% FBS (Gibco, America). TC-1 cell line was cultivated by RPMI 1640 medium with 10% FBS (Gibco, America). All cells would be incubated within a humidified box with 5% CO_2_ under a temperature of 37 th.

### Exosomal isolation and characterization

The fresh complete medium with EV-free FBS (Thermo, America) was used to incubate A549 cells for 48 h. Thereafter, the cells underwent centrifugation at 1500 g and 4 °C for 15 min. After filtration by a 0.22 mm filter (Millipore, America), the supernatant underwent two sequential centrifugations at a high speed of 110,000 g and a temperature of 4 °C for 1 h. A549-Exos were then resuspended in an appropriate volume of phosphate-buffered saline (PBS) and kept at –80 °C for subsequent application. The morphological feature of A549-Exos was observed using transmission electron microscopy. A Nano-z90 Nanosizer (Malvern, UK) was allied to evaluate the size distribution of A549-Exos. Western blot method was allied to analyze the expressional levels of exosomal surface markers such as CD9 and TSG101.

### A549-Exos^miR-328 inhibitor^ isolation

A549 cells (1 × 10^5^) were inoculated into 12-well plates and cultivated overnight. Subsequently, RNA transfection into A549 cells were performed with Lipofectamine RNAiMAX (Thermo, America) based on operational manual. These cells were incubated with fresh complete medium comprising EV-free FBS (Thermo, America) for 48 h. Then, the medium was harvested and centrifuged at 1500 g for 15 min at 4 °C. After filtration by a 0.22 mm filter (Millipore, America), the supernatant underwent two sequential centrifugations at high speed of 110,000 g and a temperature of 4 °C for 1 h. A549-Exos^miR-328 inhibitor^ were then resuspended in an appropriate volume of PBS and kept at –80 °C for subsequent application.

### Reagents

miR-328 mimics, miR-328 inhibitors and respective negative controls, Nrp-2 inhibitors and respective negative controls were provided by Shanghai GenePharma Co., Ltd, China.

### Western-blot assay

Cells or A549-Exos were immersed in RIPA solution comprising protease inhibitor cocktail (Cell Signaling Technology, America). Then, BCA Protein Assay Kit (Thermo, America) was applied to quantitatively measure the total protein concentration. A Western-blot assay was conducted according to the method in the literature [30]. The following primary antibodies were applied in the present study: CD9, HSP70, GAPDH and osteoprotegerin (OPG) protein were bought from the Cell Signaling Technology, America. All secondary antibodies (1:5000) were provided by Abcam, America.

### In vitro osteoclastogenesis of RAW 264.7 cells

RAW 264.7 cells (1 × 10^4^) were inoculated into 48-well plates in DMEM containing 10% FBS (Gibco, America). The osteoclast maturation was enhanced for 7 d by using M-CSF (30 ng/ml) and RANKL (100 ng/ml). Thereafter, TRAP staining was conducted based on operational manual. TRAP-positive cells (≥5 nuclei) were recognized as mature osteoclasts.

### Luciferase reporter assay

The mouse Nrp-2 mRNA 3′- UTR sequence, including the binding site of miR-328 binding site, was amplified to generate Nrp-2 3′-UTR luciferase reporter constructs using PCR method. Then, PCR products were screened and inserted immediately into the XbaI site of the pGL3-promoter vector (Promega, America), downstream of the stop codon of luciferase. Nrp-2 mutants were prepared with Quick-Change Site-Directed Mutagenesis Kit (Stratagene, CA) for obtaining MUT-pGL3-Nrp2, which could be identified by sequence analysis. RAW 264.7 cells firstly received transfection of WT or mutant pGL3 construct and then received transfection of 40 ng pRL-TK Renilla-luciferase plasmid, miR-328 mimics or inhibitors respectively for 48 h. Luciferase activity was detected according to the method in the literature [30].

### Real-time PCR

Total RNA extraction from RAW 264.7 cells or A549-Exos was performed with TRIzol reagent (Life Technologies, America), subsequently complementary DNA synthesis was conducted. Thereafter, quantitative reverse transcriptase PCR (qRT–PCR) was conducted according to the method in the literature [30]. For the qRT-PCR analysis, the following primers were applied: miR-328: forward, 5′-CGG GCC TGG CCC TCT CTG CC-3′; reverse, 5′- CAG CCA CAA AAG AGC ACA AT-3′; Ctsk: forward, 5′-CTC GGC GTT TAA TTT GGG AGA-3′; reverse, 5′-TCG AGA GGG AGG TAT TCT GAG T-3′; TRAF-6: forward, 5′-AAG GTG GTG GCG TTA TAC TGC-3′; reverse, 5′-CTG GCA CAG CGG ATG TGA G-3′; and GAPDH: forward, 5′-AAT GGA TTT GGA CGC ATT GGT-3′; reverse, 5′-TTT GCA CTG GTA CGT GTT GAT-3′.

### Animal studies

The treatment of animals conformed to the National Institute of Health Guide for the Care and Use of Laboratory Animals (the Guide), and had obtained an ethics approval from Longhua Hospital (No. LHERAW-19038). 6-8-week-old C57BL/6 mice came from the Animal Center of Longhua Hospital, wherein all mice were bred in hygienic environments, which provide a barrier between the mice. The mice were intravenously administered with A549-Exos or A549-Exos^miR-328 inhibitors^ (100 ng/ml in 1 ml PBS) via the tail vein once every three days for four weeks (*n* = 7 in each group). The mice were killed at four weeks after the procedure.

### Micro-CT examination

Femur specimen harvested from the mice underwent scanning and analysis using Quantum GX micro-CT (PE, America). All micro-CT scanning was conducted under the following conditions: voltage 100 kV, current 80 μA, spatial resolution 12 μm, and 500 continuous sections were scanned. The Skyscan CT-analyzer was used for automated analysis of the number of trabecular bones (Tb.N), trabecular bone thickness (Tb.Th), trabecular bone space (Tb.Sp), bone volume fraction (BV/TV) and three-dimensional reconstruction.

### Exosome staining and tracking in vivo

A549-Exos were stained to visualize the target organ and the metabolic process of Exos based on the instruction manual (Thermo, America). First, 100 µg Exos were stained using Vybrant DID (1:1000 in PBS) in darkness for 30 min, subsequently the stained Exos were rinsed with PBS and underwent centrifugation at a speed of 100,000 × *g* for 60 min. Thereafter, three 8-week-old mice were intravenously administrated with the stained Exos via the tail vein. The mice were harvested at 3, 6, and 12 h after the treatment. The average intensities of the DID signals were quantitatively detected in 4 random view fields per section.

### HE & TRAP staining

We dissected femora from the mice in different groups to carry out histological staining, the femora underwent 4% paraformaldehyde fixation for 24 h, followed by EDTA (ethylene diamine tetraacetic acid pH = 7.4) decalcification for 28 d, ethanol dehydration and paraffin embedding. Then, the longitudinal sections of femora with a thickness of 10 μm were prepared and stained with haematoxylin-eosin or for TRAP according to the method in the literature [30].

### Statistical analysis

The data were expressed as average value with standard deviations (SD), and all experiments were conducted in triplicate. *t* test and one-way ANOVA were used for comparison between groups as appropriate. A *P* value less than 0.05 was accepted as statistically significant.

## Supplementary information

Figure S1

Figure S2

Figure S3

Figure S4

supplementary legends

WB data
