# Supplementary figures and images for: Exosomal miR-328 originated from pulmonary adenocarcinoma cells enhances osteoclastogenesis via downregulating Nrp-2 expression
Source: Cell Death Discov. 2022 Oct 3;8:405. doi: 10.1038/s41420-022-01194-z (PMC9530222; doi:10.1038/s41420-022-01194-z)

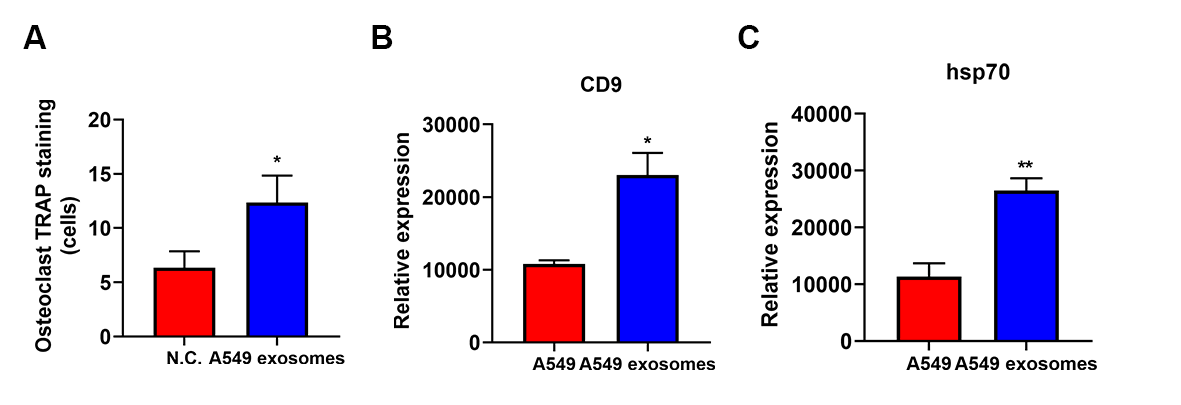

Supplement: Supplementary file 1 — Figure S1 [file 41420_2022_1194_MOESM1_ESM.tif]

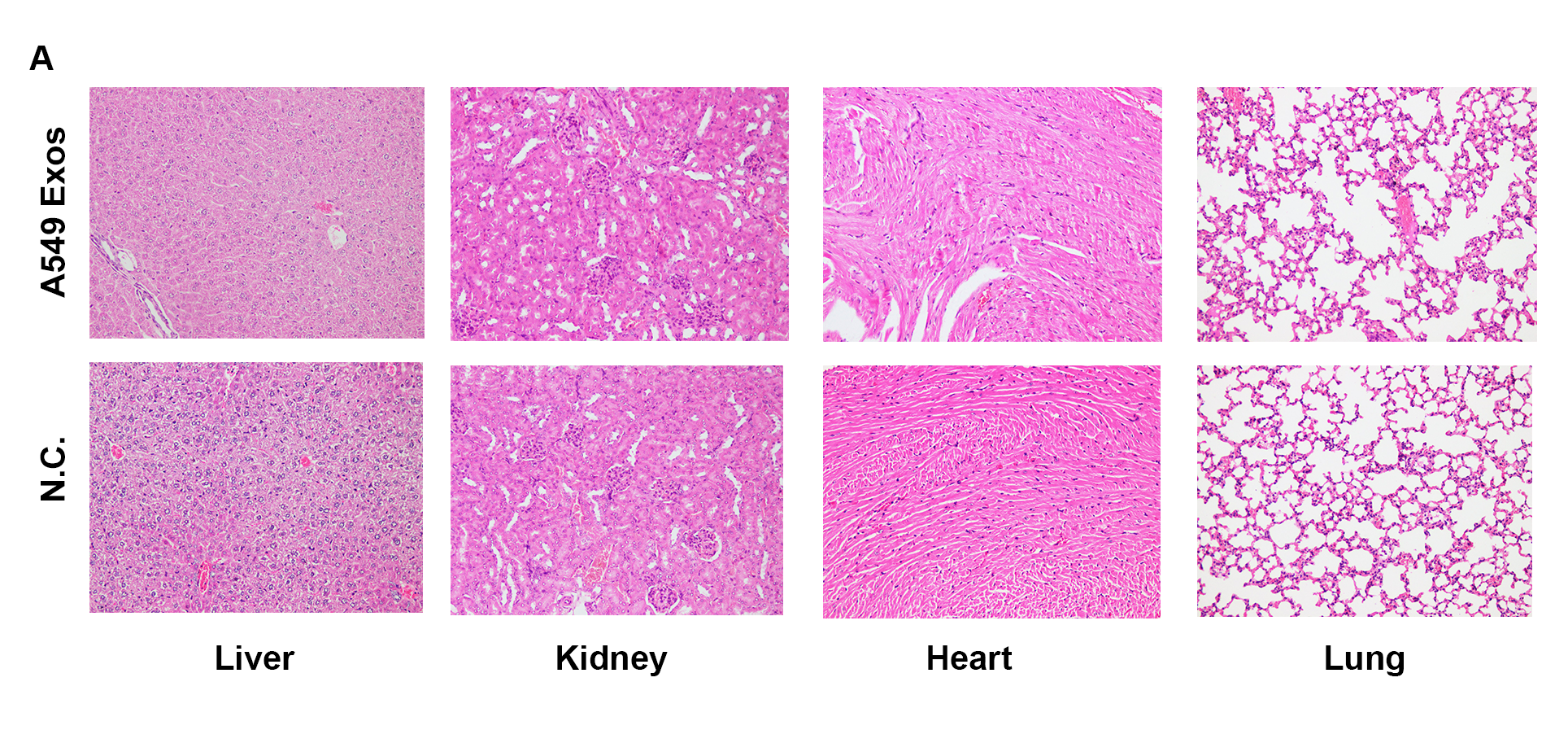

Supplement: Supplementary file 2 — Figure S2 [file 41420_2022_1194_MOESM2_ESM.tif]

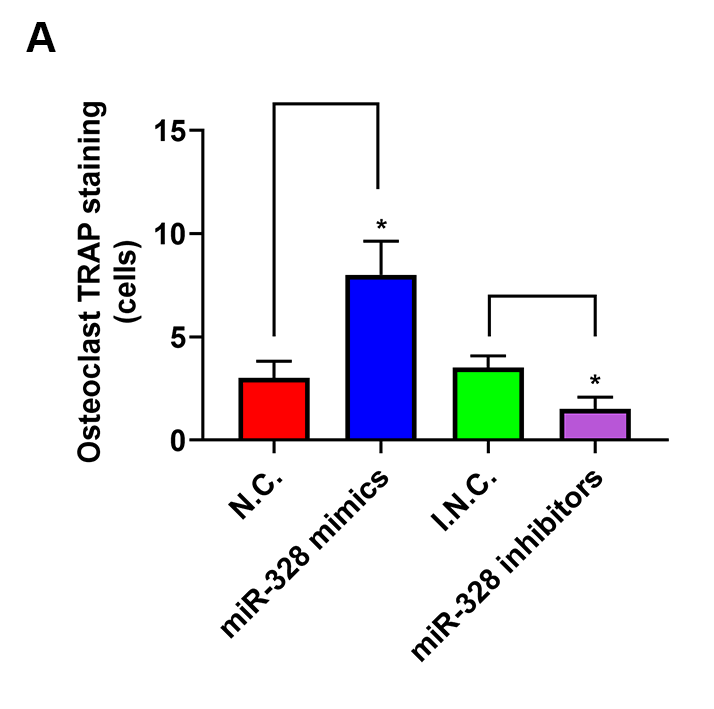

Supplement: Supplementary file 3 — Figure S3 [file 41420_2022_1194_MOESM3_ESM.tif]

Figure 1

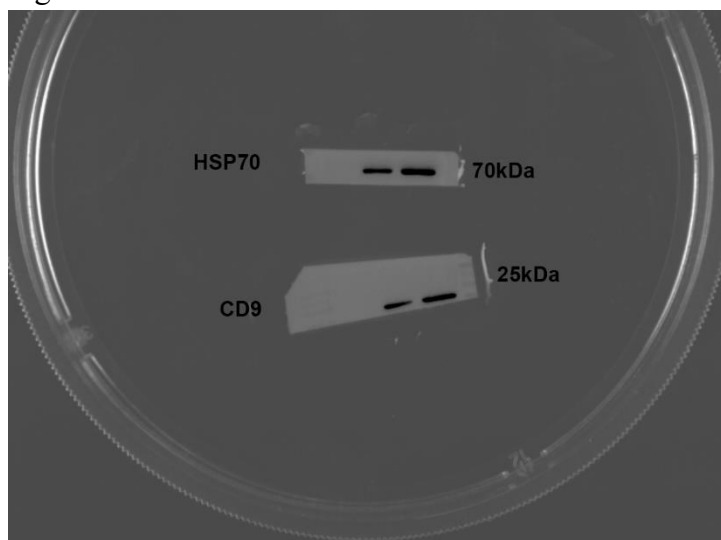

GAPDH

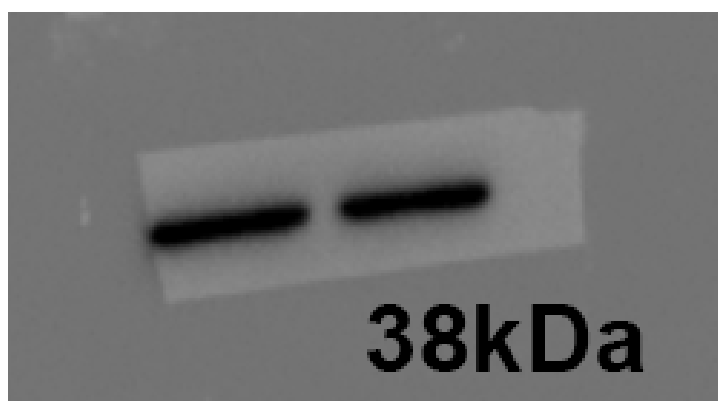

Figure 5  
GAPDH

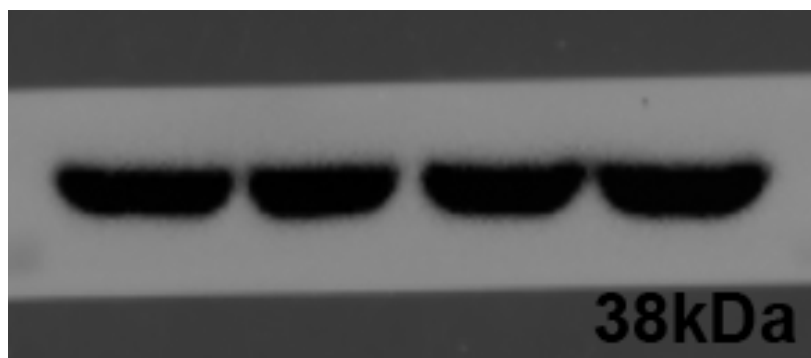

OPG

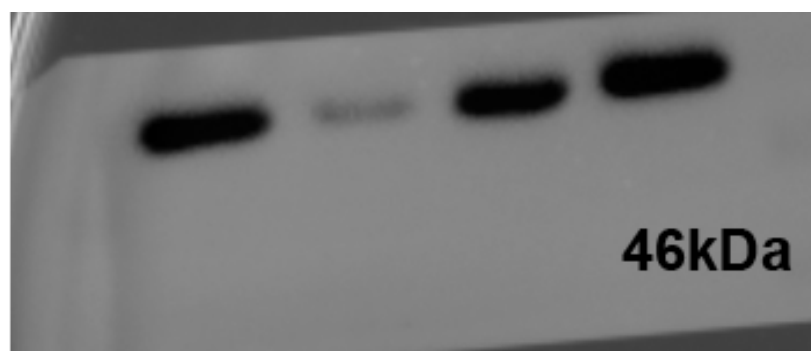

Supplement: Supplementary file 6 — WB data [file 41420_2022_1194_MOESM6_ESM.pdf]
