# Supplementary material for: Exosomal miR-328 originated from pulmonary adenocarcinoma cells enhances osteoclastogenesis via downregulating Nrp-2 expression
Source: Cell Death Discov. 2022 Oct 3;8:405. doi: 10.1038/s41420-022-01194-z (PMC9530222; doi:10.1038/s41420-022-01194-z)
Supplement: Supplementary file 5 — supplementary legends [file 41420_2022_1194_MOESM5_ESM.docx]

**Figure S1 (A).** Quantitative analysis of positive TRAP-stained osteoclasts is shown in Figure 1A. (B). Quantification analysis of CD9 and HSP70 by a Western blot analysis. **P* < 0.05, ***P* < 0.01.

**Figure S2 (A).** There were no histomorphometric changes in the heart, liver, lung or kidney.

**Figure S3** (A). Quantitative analysis of positive TRAP-stained osteoclasts is shown in Figure 5D. **P* < 0.05.

**Figure S4** (A). Representative images of TRAP staining of mature osteoclasts treated with N.C. and Trp2 inhibitors. (B). Quantitative analysis of positive TRAP-stained osteoclasts is shown in Figure S4A. **P* < 0.05.
